# Confronting species distribution model predictions with species functional traits

**DOI:** 10.1002/ece3.1898

**Published:** 2016-02-22

**Authors:** Marion E. Wittmann, Matthew A. Barnes, Christopher L. Jerde, Lisa A. Jones, David M. Lodge

**Affiliations:** ^1^ Department of Biological SciencesUniversity of Notre DameNotre DameIndiana46556; ^2^ Department of BiologyUniversity of Nevada RenoRenoNevada89509; ^3^ Environmental Change InitiativeUniversity of Notre DameNotre DameIndiana46556; ^4^ Department of Natural Resources ManagementTexas Tech UniversityLubbockTexas79409; ^5^ Fisheries and Oceans CanadaGreat Lakes Laboratory for Fisheries and Aquatic SciencesBurlingtonON L7S 1A1Canada

**Keywords:** Environmental niche model, grass carp, managed species, Maxent, model validation, species biogeography, species functional traits

## Abstract

Species distribution models are valuable tools in studies of biogeography, ecology, and climate change and have been used to inform conservation and ecosystem management. However, species distribution models typically incorporate only climatic variables and species presence data. Model development or validation rarely considers functional components of species traits or other types of biological data. We implemented a species distribution model (Maxent) to predict global climate habitat suitability for Grass Carp (*Ctenopharyngodon idella*). We then tested the relationship between the degree of climate habitat suitability predicted by Maxent and the individual growth rates of both wild (*N* = 17) and stocked (*N* = 51) Grass Carp populations using correlation analysis. The Grass Carp Maxent model accurately reflected the global occurrence data (AUC = 0.904). Observations of Grass Carp growth rate covered six continents and ranged from 0.19 to 20.1 g day^−1^. Species distribution model predictions were correlated (*r = *0.5, 95% CI (0.03, 0.79)) with observed growth rates for wild Grass Carp populations but were not correlated (*r = *−0.26, 95% CI (−0.5, 0.012)) with stocked populations. Further, a review of the literature indicates that the few studies for other species that have previously assessed the relationship between the degree of predicted climate habitat suitability and species functional traits have also discovered significant relationships. Thus, species distribution models may provide inferences beyond just where a species may occur, providing a useful tool to understand the linkage between species distributions and underlying biological mechanisms.

## Introduction

Understanding the distribution of species and ecosystems as well as the underlying biological mechanisms is essential to the sustainable management of natural resources. The study of biogeography, however, has largely developed separately from ecosystem ecology, which has led to conceptual and technical difficulties in incorporating species interactions, dispersal limitations, and species' adaptations into predictive models (Violle et al. 2014). Functional components of biodiversity, for example, the distribution of species forms and functions, have recently been recognized as important linkages between observed species distributions and the associated abiotic and biotic conditions of ecosystems (Wardle et al. 2004; Violle et al. 2014). The study of these relationships has also benefitted applications in conservation biogeography and other fields (Franklin 2010, Griffith et al. 2016).

Observed species distributions are the result of the abiotic and biotic conditions and processes affecting the species. Most of the tools that have been developed to model species distributions rely on the concept of the environmental niche and thus focus on the abiotic conditions affecting species distributions (Busby 1991; Guisan and Zimmermann 2000; Phillips et al. 2006). Termed “environmental niche model” or SDM (“species distribution model”), these predictive models combine known occurrences of a species with local environmental data (often, climate‐based data such as temperature or precipitation) to predict potential species geographic distribution (i.e., the “fundamental niche” (Hutchinson 1958) including areas where the species is known to occur as well as areas where it does not). SDMs have been used in a wide range of applications such as habitat selection for species introductions and conservation (Schwartz et al. 2012), predicting invasive species spread (Jiménez‐Valverde et al. 2011; Sobek‐Swant et al. 2012), and estimating response to global climate change (Guisan and Thuiller 2005). An implicit assumption of SDMs is that in sites predicted to be highly suitable, species would have higher fitness compared to sites predicted to be poorly suitable (Guisan and Thuiller 2005); however, this relationship is rarely tested.

Despite demonstrations of SDM accuracy in predicting species occurrence (e.g., Chen et al. 2006; Herborg et al. 2007), skepticism remains about how accurate we might expect predictions to be given the lack of ecological or biological information in most SDM applications. First, because SDMs are based on the concept of the environmental niche and focus on the abiotic conditions affecting species distributions, model outputs are more representative of the potential species distribution, rather than the realized or observed species distribution that has been shaped by biotic conditions and ecological processes. Second, niche conservatism, a major tenet of ecological niche modeling of nonindigenous species, hypothesizes that a species will spread primarily into areas within which its climatic niche is similar to that of its native range (Pearman et al. 2008). However, counter‐examples exist (Broennimann and Guisan 2008; Tingley et al. 2014), potentially due to ecological or evolutionary niche shifts, landscape heterogeneity, model selection, choice of environmental variables used to train models (Peterson and Nakazawa 2007; Rödder and Lötters 2009, Teller et al. 2016), or interspecific interactions (Sinclair et al. 2010). Further, historical conditions also influence observed species distributions and can make predicting distributions problematic using only environmental variables.

For one widely distributed species, we tested whether climate habitat suitability predictions resulting from Maxent analyses are correlated with observations of growth rate. Specifically, we used Grass Carp (*Ctenopharyngodon idella*), a widely distributed aquaculture and nuisance aquatic plant control species (but also an invasive species in some regions and ecosystems), to test whether the degree of predicted climate habitat suitability correlates positively with observations of individual growth rate.

## Methods

### Model organism

Grass Carp is a large cyprinid fish with a native range extending from northern Vietnam to the Amur River along the Russia–China border (Fuller et al. 1999). It has been widely introduced for nuisance aquatic plant control and is also cultivated in China and other countries worldwide as a food source. Recently in North America, concern about its persistence and potential unwanted impacts has increased because of a growing number of captures of feral individuals in unintended locations (Wittmann et al. 2014).

### Species distribution model implementation

We predicted Grass Carp climate habitat suitability at the global scale using Maxent because in many applications it has better performance than other SDM methods and because it is the most widely used SDM software implementation (Elith et al. 2006; Phillips et al. 2006; Fitzpatrick et al. 2013). Full details of Maxent implementation are presented in Appendix S1, including a description of occurrence data preparation, environmental data, and the results of a pilot tuning experiment. Briefly, we rarified Grass Carp occurrence data and incorporated bias grids (Elith and Kearney 2010) to avoid reporting biases, which could influence model performance (Barnes et al. 2014). We used only temperature layers (omitting precipitation layers) of the WorldClim climate data set (Hijmans et al. 2005) as the source of environmental data for this study. As an aquatic species, Grass Carp establishment can occur only in aquatic habitats. Adapting SDM implementation methods used in (Barnes et al. 2014), we did not include any indicators of water availability in the environmental layers used to train our models because even in regions where standing water is not plentiful, such as the southwestern United States, Grass Carp could establish if introduced into riverine backwaters, oases, or water gardens, and we did not want our model to miss suitable habitat in such areas. Model performance was assessed directly in our pilot tuning experiment through iterative omission of random subsets of 20% of occurrence data for testing of predictive strength using area under the receiver operating characteristic curve (AUC). To maximize data availability for the main purpose of this study – the comparison of Maxent output with Grass Carp growth data – we ran Maxent with all available occurrence data. We present only this all‐data model in the main text.

### Grass Carp growth rate

Data to summarize observed growth rates of Grass Carp were obtained from the primary and gray literatures using ISI Web of Science and Google Scholar keyword search terms: “grass carp” and “*Ctenopharyngodon idella*”. Studies were retained if they quantified growth rates of Grass Carp under natural conditions (either wild or naturalized populations) (termed “wild” below) or under conditions in which Grass Carp were stocked for nuisance plant control and the experimental period in which fish were monitored was as least 6 months (termed “stocked” below). Studies originally published in Russian and not available in English were translated (Bogutskaya et al. In Press). Situations in which Grass Carp were artificially fed, supplemented (e.g., protein pellets or other non‐natural food sources such as terrestrial plants or feed), or were from artificial tanks, mesocosms, or laboratory enclosures were excluded to avoid bias in growth rates associated with non‐natural habitat or feeding conditions. Growth was calculated in grams per day (g day^−1^). In cases where there were multiple age classes or cohorts, growth rates were averaged over all classes. See Appendix S2 for a full list of all observations used.

### Correlation analyses

We used Pearson's correlation to assess the relationship between Grass Carp individual growth rate and the predicted degree of climate suitability from Maxent (i.e., the logistic output of the Maxent model). Two independent tests were performed on the relationship between predicted climate habitat suitability and individual growth rate: growth rates from wild captures and growth rates for stocked Grass Carp. The null hypothesis was that no correlation exists between observed growth rate and climate habitat suitability, *r *=* *0. We hypothesized a positive, significant relationship, especially for the wild populations. We expected the relationship to be weaker or nonexistent for the stocked Grass Carp because they may have high and/or rapidly changing resource abundance (e.g., if stocked to reduce or eliminate nuisance macrophytes) or they may experience unusually large densities as part of the initial stocking conditions, such that density dependence may restrict individual growth. Because density of stocked populations, and age of both stocked and wild populations, may be correlated with growth rate, we also tested the correlation of density with growth rate for each age class with available data. These last two tests were carried out to avoid spurious correlations between growth rate and climate habitat suitability. Hypothesis testing is reported using 95% confidence intervals of the correlation coefficient.

### Literature review

We conducted a literature review to summarize other documented examples of the relationship between species functional traits and species distribution model outputs. Data were obtained from the primary literature using ISI Web of Science and Google Scholar searches for studies that assessed the relationship between any kind of species distribution model (not just Maxent but also boosted regression trees, generalized additive models, and others). Search terms used included “species distribution model” and “environmental niche model”. Information collected from each study included organism type, species, location, model(s) used, traits evaluated, relationship (positive, negative, or none), and reference information.

## Results

### Species distribution model

**Figure 1:**
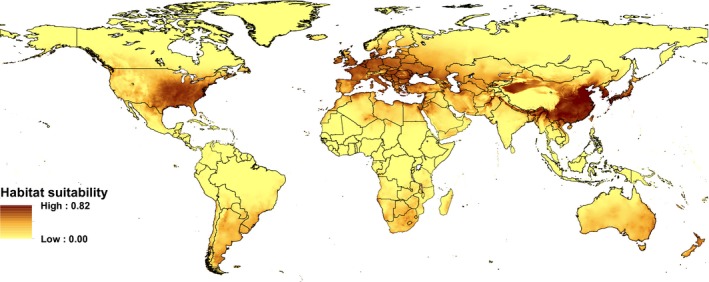
Global projection of suitable Grass Carp (*Ctenopharyngodon idella*) habitat based on occurrences records with spatial extent of 50 km or less. Shading indicates the logistic output of the model. See Appendix S2 for Grass Carp occurrence records.

Overall, Maxent accurately captured the known global occurrences of Grass Carp (AUC = 0.904). Predicted highly suitable habitat occurred within Grass Carp native range in eastern Asia between the Amur River and the northern regions of the Cambodian peninsula (Fig. 1). Similar areas of high suitability occurred in coastal regions of Australia and the coasts of the Mediterranean Sea, southeastern regions of North America, including large portions of the United States and Mexico, as well as southern Brazil and large portions of Uruguay, Paraguay, and Argentina in South America. Predicted climate habitat suitability was low along the equator and north of the Arctic Circle (Fig. 1).

### Grass carp growth rate

We found 68 unique records from six continents of Grass Carp growth rate that ranged from 0.19 to 20.1 g day^−1^ (Appendix S2). Seventeen of 68 records were considered wild or feral populations and were observed in Russia, Kazakhstan, Uzbekistan, Turkmenistan, New Zealand, and the United States. The remaining 51 records were widely distributed stocked Grass Carp populations measured in canals, ponds, lakes, and river or reservoir systems. Fish ages ranged from yearling to greater than 9 years.

### Correlation analysis

**Figure 2:**
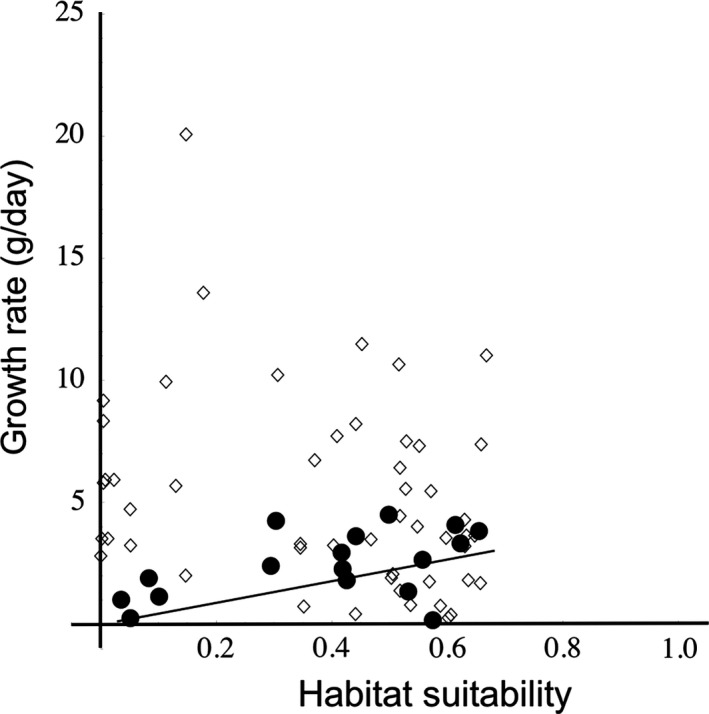
Scatterplot of growth rate (g day^−1^) and Maxent‐predicted habitat suitability of Grass Carp from stocked (*n *=* *51, open diamonds) and wild captures (*n *=* *17, black circles). Pearson's correlation coefficient testing revealed only wild captured had a significant (95% CI: 0.03, 0.79) and positive (*r *=* *0.5) correlation between growth rate and habitat suitability. The black line indicates the positive relationship of the wild population correlation.

For observed stocked populations, neither the stocking density (*r *=* *−0.07, 95% CI (−0.32, 0.19), *n *=* *58), nor fish age (*r *=* *0.02, 95% CI (−0.24, 0.28), *n *=* *59) correlated with growth rate. Similarly with wild populations, age did not correlate with growth rate (*r *=* *−0.35, 95% CI (−0.76, 0.25), *n *=* *13), giving us confidence that any correlations between the degree of climate habitat suitability and growth are not spurious. For stocked Grass Carp populations, the correlation of growth rate and degree of climate habitat suitability was not significant (*r = *−0.26, 95% CI (−0.5, 0.012), *n *=* *51). However, as predicted, the correlation for wild Grass Carp was positive and significant (*r = *0.5, 95% CI (0.03, 0.79), *n *=* *17) (Fig. 2).

### Literature review

Few studies in the published literature compared species distribution model estimates with species functional trait observations (Table 1). We found four published studies that evaluated 22 species including evergreen trees (*n* = 1 species), grassland plants (*n* = 4), common alpine plants (*n* = 16), and crayfishes (*n* = 1). There were nine species functional traits evaluated including genetic diversity, asymmetry, leaf weight, fecundity, and others (Table 1). Nineteen of 22 species evaluated had significant positive or negative relationships between SDM output and measured functional trait.

**Table 1:** Summary of studies showing relationship between variation in habitat suitability and species functional traits. Organism type, species name, region in which relationships were tested, modeling platform used, and specific traits evaluated are given in columns 1–5. Relationships between traits evaluated by trait and/or by species indicated in column 6 (Relationship): +, positive relationship; −, negative relationship; 0, no relationship. Study reference given in last column. ^†^Number of species with habitat‐specific relationships determined

Organism(s)	Species	Location	Model(s) used	Traits evaluated	Relationship	Reference
Evergreen Tree	*Myristica malabarica*	Western Ghats, India	Bioclim (DIVA GIS v 7.3)	Regeneration ability	+	Nagaraju et al. (2013)
			Maxent (v 3.3.2)	Genetic diversity	+	
				Fluctuating asymmetry	+	
				Specific leaf weight	+	
Grassland plants	*Bromus madritensis*	Coastal California, USA	Boosted Regression Tree (R v 2.3.1)	Fecundity	+	Elmendorf and Moore (2008)
	*Geranium dissectum*		Artificial Neural Network (R v 2.3.1)		0	
	*Lupinus nanus*				0	
	*Vulpia microstachys*				+	
Common Alpine Plants	*Carex sempervirens*	Central French and Western Swiss Alps	Generalized Additive Model (R v 2.8.2)	Leaf dry matter content	− (17/21)^†^ + (4/21)	Thuiller et al. (2010)
	*Dactylis glomerata*					
	*Dryas octopetala*					
	*Festuca paniculata*					
	*Geum montanum*					
	*Juniperus* sp.			Leaf Nitrogen content	− (15/21)^†^ + (6/21)	
	*Larix deciduas*					
	*Leucanthemum vulgare*					
	*Pinus* sp.					
	*Polygonum viviparum*					
	*Rhododendron ferrugineum*			Maximum vegetative height	− (12/21)^†^ + (9/21)	
	*Sesleria caerulea*					
	*Salix herbacea*					
	*Silene nutans*					
	*Trifolium alpinum*					
	*Vaccinium myrtillus*					
Crayfish	*Pacifastacus leniusculus*	Pacific Northwest, Japan	Maxent (v 3.3.3e)	Trophic position (*δ* ^13^C)	0	Larson et al. (2010), Larson pers comm
Freshwater fish	*Ctenopharyngodon idella* (stocked)	Global	Maxent (v 3.3.3k)	Growth rate	0	This study
	*Ctenopharyngodon idella* (wild)				+	

## Discussion

Our model species, Grass Carp, provided an opportunity to test hypotheses concerning SDM predictions on a global scale. Because of the widespread distribution of Grass Carp, and its status as both a beneficial (e.g., as a stocked species for food and/or biocontrol) and nuisance species (e.g., when feral populations have unwanted impacts to ecosystems) data exist worldwide related to its occurrence and growth. This is in contrast to most other species where observations of species functional traits, such as growth, are limited to small empirically based laboratory or field settings. Although the sample size of wild grass carp populations is small (*n *=* *17), the records are the best available information of georeferenced measurements of grass carp growth rates outside of manipulated or stocked situations, and represent independent populations.

For stocked populations of Grass Carp, the lack of correlation between habitat suitability and growth rates was expected, and the reasons for this may be relevant to some applications of SDM to other nonindigenous species. One assumption of ecological niche theory typically disregarded in SDM implementation is that species distributions are static in space and time, that is, the species occurrence is in equilibrium with its environment (Guisan and Thuiller 2005). During range expansion, however, populations of nonindigenous species are not at equilibrium if dispersal limitation exists or sink populations occur (Dullinger et al. 2009, Uriarte et al. 2016). It is possible that the stocked populations of Grass Carp were in flux with their environment due to an initially high abundance of macrophytes that may decline over time as biocontrol populations have the desired impact. Other Grass Carp‐induced changes in the environment, such as altered turbidity, nutrient concentrations, or species dynamics may also cause growth rates to change over time. Additionally, it is possible that a number of the stocked grass carp population were diploid, triploid, or a mix of both, potentially affecting their growth. Previous work has indicated that triploid and diploid grass carp have similar growth rates (Wiley and Wike 1986). However, it has also been shown that diploid grass carp have higher growth rates when in the presence of triploid grass carp (Cassani and Caton 2011). Here, the lack of both population‐specific information on grass carp ploidy and a relationship for stocked populations reinforces the need for caution in analyses of dynamic populations in flux (Henning‐Lucass et al. 2016, Visser et al. 2016).

Only a few studies have assessed the relationship between the degree of SDM predicted climate habitat suitability and species functional traits (Table 1). These previous results indicate that correlations between climate habitat suitability model outputs and species traits exist, but vary by species type, landscape, and functional trait. Differences in results among studies have been attributed to dynamics occurring with individuals, communities, or micro‐habitats (e.g., adaptation, disturbance, community composition, and variability in abiotic response) (Elmendorf and Moore 2008; Larson et al. 2010; Thuiller et al. 2010; Nagaraju et al. 2013).

Our study adds the first fish example to this growing body of support that SDM outputs can indicate more than simply potential range extent and/or densities (Oliver et al. 2012) of species' ranges and may capture species functional traits, such as growth rate, which may be an indicator of fitness. However, the evidence to support the notion that the degree of habitat suitability predicted by SDM applies to biological performance in addition to potential occurrence remains sparse and should be expanded in future SDM studies (Gallien et al. 2010). We are not suggesting that at this point SDM estimates may be used to infer traits. However, if more studies demonstrate that climate habitat suitability is correlated with species functional traits, such as growth rate, then ecology, conservation biology, aquaculture, and other applications may benefit from future SDM efforts.

These results represent a call for increased diligence in producing climate habitat suitability models and utilizing them to evaluate the relationship between species functional traits and distributions. With so much effort being put into the refinement of modeling approaches on the one hand, and criticism of methodological assumptions and initial conditions on the other hand, there has been insufficient attention to evaluating the biological meaning of SDM output. Confronting the output of SDMs with biological performance data can provide new analyses with which to evaluate limitations and/or new potential uses of SDM. If correlations between climate habitat suitability and other biological factors exist, then ecologists and spatial scientists can be better positioned to offer broader inferences from SDMs beyond where species may occur.

## Conflict of Interest

None declared.

## Supporting information


**Appendix S1.** Detailed methods of Maxent implementation.

**Appendix S2.** Grass Carp growth rate occurrences, growth rate occurrence descriptive table and associated references.
