# Supplementary material for: Confronting species distribution model predictions with species functional traits
Source: Ecol Evol. 2016 Feb 22;6(4):873–9. doi: 10.1002/ece3.1898 (PMC4761765; doi:10.1002/ece3.1898)
Supplement: Supplementary file 1 — Appendix S1. Detailed methods of Maxent implementation. [file ECE3-6-0873-s001.docx]

**SOM A – Detailed methods of Maxent implementation**

**Occurrence Data Preparation**

Grass Carp (*Ctenopharyngodon* *idella*) locality information (i.e., positive occurrence) throughout the globe was obtained from the primary literature and the Global Biodiversity Information Facility (gbif.org), Fishbase (fishbase.org; Froese and Pauly 2011), and USGS (usgs.nas.gov) databases. Each georeferenced position was verified, and error radii were assigned using MaNIS (Wieczorek, Guo & Hijmans 2004). All localities with an uncertainty of position >50km were removed to reflect the scale of the environmental data. Only observations of Grass Carp that were either recorded live or captured from a pond, lake, river, reservoir or other waterbodies were included. Observations from outdoor markets or other commercial sources were not included in this analysis. This dataset included details from USGS that identified populations as “established,” which was defined as a population that is reproducing and overwintering (USGS 2012). Overall, 663 Grass Carp occurrence records were collected (Figure A1).
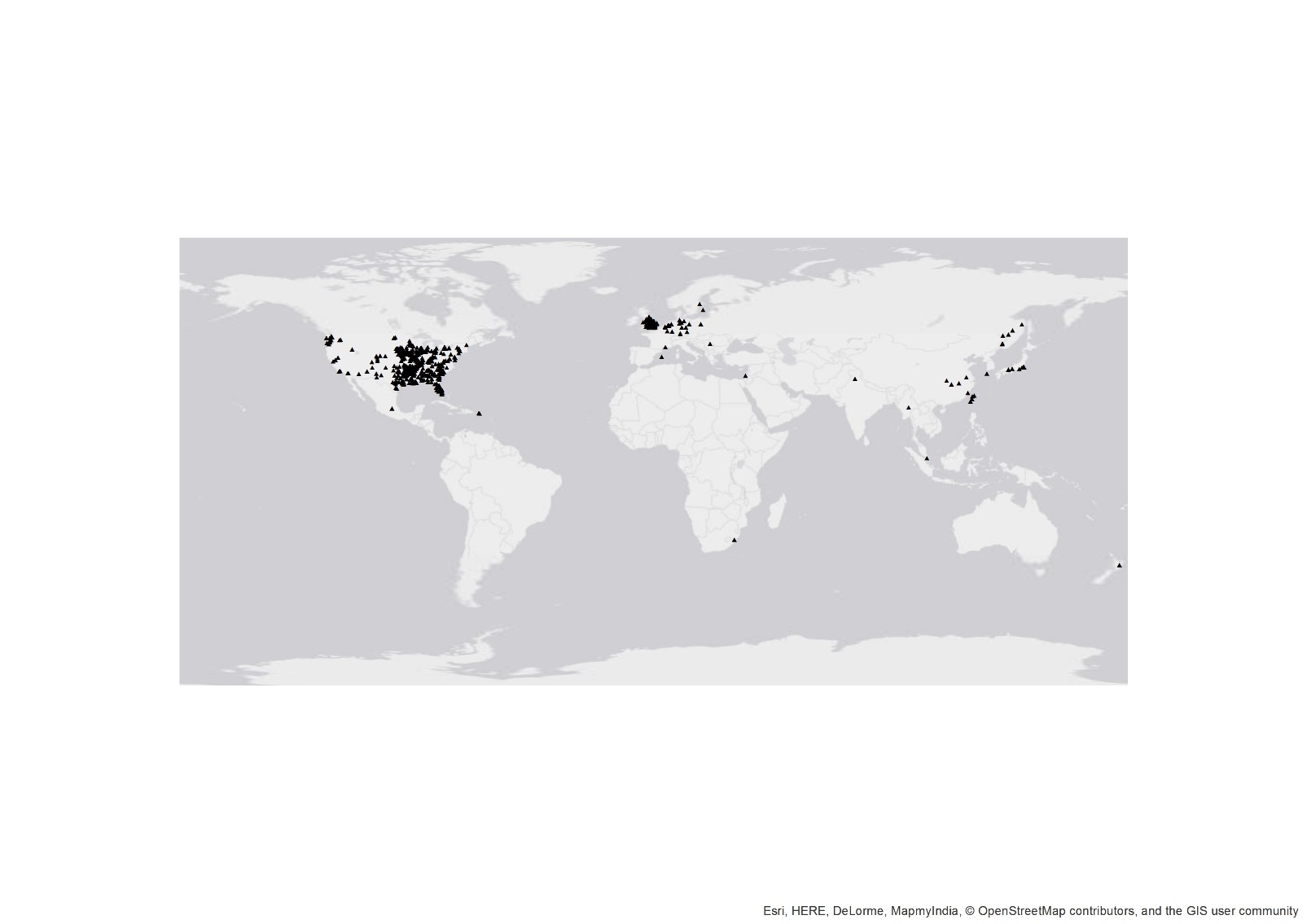


Figure A1. Grass Carp occurrence records (▲; *n*=663) were recovered from across the globe for development of a species distribution model with Maxent.

The environmental variable dataset was comprised of BioClim layers, which are derivations of global temperature and precipitation data presumed to be generally relevant to construction of abiotic niche space (Hijmans et al. 2005; worldclim.org). We used layers with a 10 arcminute resolution because this resolution was closest to the error radii described above for screening of Grass Carp occurrence data. Because Grass Carp are obligate aquatic organisms with an extensive history of intentional stocking, we limited our environmental layers to exclude those describing precipitation and include only those describing temperature (i.e., annual mean temperature, mean diurnal range, isothermality, temperature seasonality, maximum temperature of warmest month, minimum temperature of coldest month, and temperature annual range).

Reporting bias in occurrence data influences model performance (Barnes et al. 2014), so we prepared Grass Carp occurrence data for our use by rarifying data to match the 10 arcminute scale of our environmental layers. To accomplish this task, we converted occurrence points into a raster file, then back into a points file, resulting in a maximum of one occurrence per environmental grid cell (all data preparation and model visualizations performed in ArcGIS 10.1, Environmental Systems Research Institute, Redlands, California, USA). The Grass Carp dataset prepared in this way included 403 occurrences.

Maxent also permits the inclusion of a “bias grid” which prompts the program to weigh the importance of occurrence records inversely proportional to their proximity to neighboring occurrences during model development (Elith *et al.* 2010). As an additional guard against reporting bias, we developed a global bias grid following the methods of Barnes et al. (2014). We projected rarified Grass Carp occurrences and background points (created by converting the BioClim annual mean temperature raster to points) to World Equidistant Conic projection, and we used Geospatial Modelling Environment version 0.7.3 (Spatial Ecology, LLC) to calculate pairwise distances between each occurrence and background point. We used SPSS version 22 (IBM Corporation) to calculate the Gaussian weight of each background point based on the equation

$$e^{(-(d^{2})/(2 \times s^{2}))}$$

where d = calculated distance between points and s = standard deviation of the occurrence data (50 km reflecting the error radii described above). We appended original background points with aggregated Gaussian weights in ArcGIS to create the bias grid, which we returned to WGS84 projection for use in Maxent.

**Model Parameter Tuning**

The settings used in Maxent modeling have important implications for model performance (Merow, Smith & Silander 2013). For instance, we performed a tuning experiment to select the most appropriate regularization parameter (β) for use in our model ((Phillips, Dudı & Dudik 2008; Radosavljevic & Anderson 2014). Across a range of potential regularization parameters (β = 0.02, 0.05, 0.10, 0.22, 0.46, 1.00, 2.20, 4.60, 6, 8, and 10) we developed 10 models (for each parameter) withholding a random 20% of Grass Carp occurrence data for model testing. We assessed model performance using the difference between training and testing AUC values reported by Maxent as a threshold-independent quantitative indicator (Warren & Seifert 2011; Figure A3). As a threshold-dependent quantitative metric we examined the 10 percentile training omission threshold reported within Maxent output (Radosavljevic & Anderson 2014; Figure A4). We concluded that β = 4.6 was the most reasonable value to use with our data. The average testing data AUC across the 10 models using β = 4.6 was 0.920 (± 0.006 S.D.), indicating strong predictive power.

**Figure A3. Tuning experiment found β = 4.6 approximately minimized the difference between training and testing AUC.**


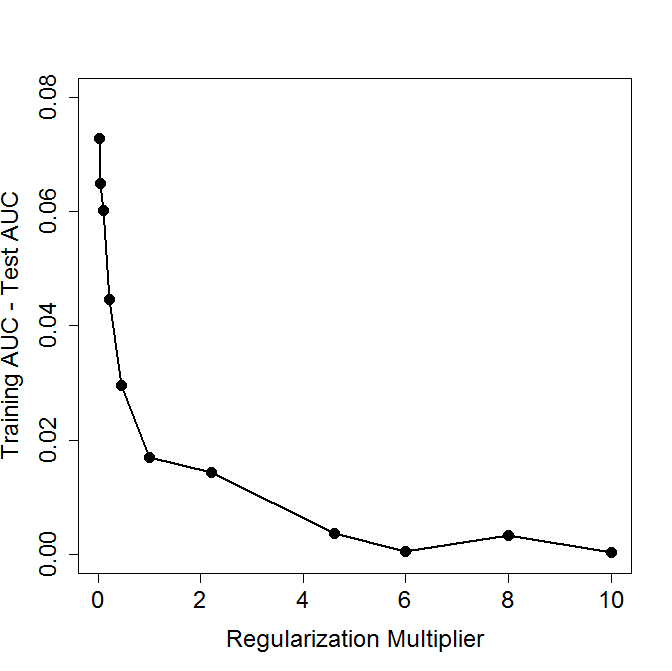


**Figure A4. Tuning experiment found β = 4.6 coincided with lowest observed omission rates.**


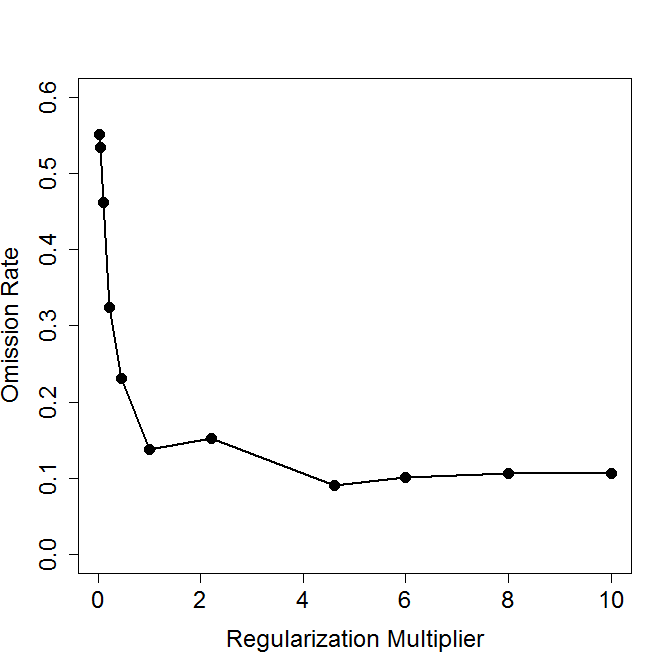


**References**

Barnes, M.A., Jerde, C.L., Wittmann, M.E., Chadderton, W.L., Ding, J., Zhang, J., Purcell, M., Budhathoki, M. & Lodge, D.M. (2014) Geographic selection bias of occurrence data influences transferability of invasive *Hydrilla verticillata* distribution models. *Ecology and Evolution*, **4**, 2584–2593.

Elith, J., Kearney, M. & Phillips, S. (2010) The art of modelling range‐shifting species. *Methods in Ecology and Evolution*, **1**, 330–342.

Froese, R. & Pauly, D. (2011) Fishbase. www.fishbase.org. Accessed 2012.

Hijmans, R.J., Cameron, S.E., Parra, J.L., Jones, P.G. & Jarvis, A. (2005) Very high resolution interpolated climate surfaces for global land areas. *International Journal of Climatology*, **25**, 1965–1978.

Merow, C., Smith, M.J. & Silander, J.A. (2013) A practical guide to MaxEnt for modeling species’ distributions: what it does, and why inputs and settings matter. *Ecography*, **36**, 1058–1069.

Phillips, S.J., Dudı, M. & Dudik, M. (2008) Modeling of species distributions with Maxent: New extensions and a comprehensive evaluation. *Ecography*, **31**, 161–175.

Radosavljevic, A. & Anderson, R.P. (2014) Making better Maxent models of species distributions: complexity, overfitting and evaluation (ed M Araújo). *Journal of Biogeography*, **41**, 629–643.

USGS. (2012) Nonindigenous Aquatic Species Database. http://nas.er.usgs.gov. Accessed 2012.

Warren, D.L. & Seifert, S.N. (2011) Ecological niche modeling in Maxent: the importance of model complexity and the performance of model selection criteria. *Ecological Applications*, **21**, 335–342.

Wieczorek, J., Guo, Q. & Hijmans, R. (2004) The point-radius method for georeferencing locality descriptions and calculating associated uncertainty. *International Journal of Geographical Information Science*, **18**, 745–767.
