# Supplementary material for: Confronting species distribution model predictions with species functional traits
Source: Ecol Evol. 2016 Feb 22;6(4):873–9. doi: 10.1002/ece3.1898 (PMC4761765; doi:10.1002/ece3.1898)
Supplement: Supplementary file 2 — Appendix S2. Grass Carp growth rate occurrences, growth rate occurrence descriptive table and associated references. [file ECE3-6-0873-s002.docx]

SOM B - Grass Carp growth rate occurrences, growth rate occurrence descriptive table and associated references.


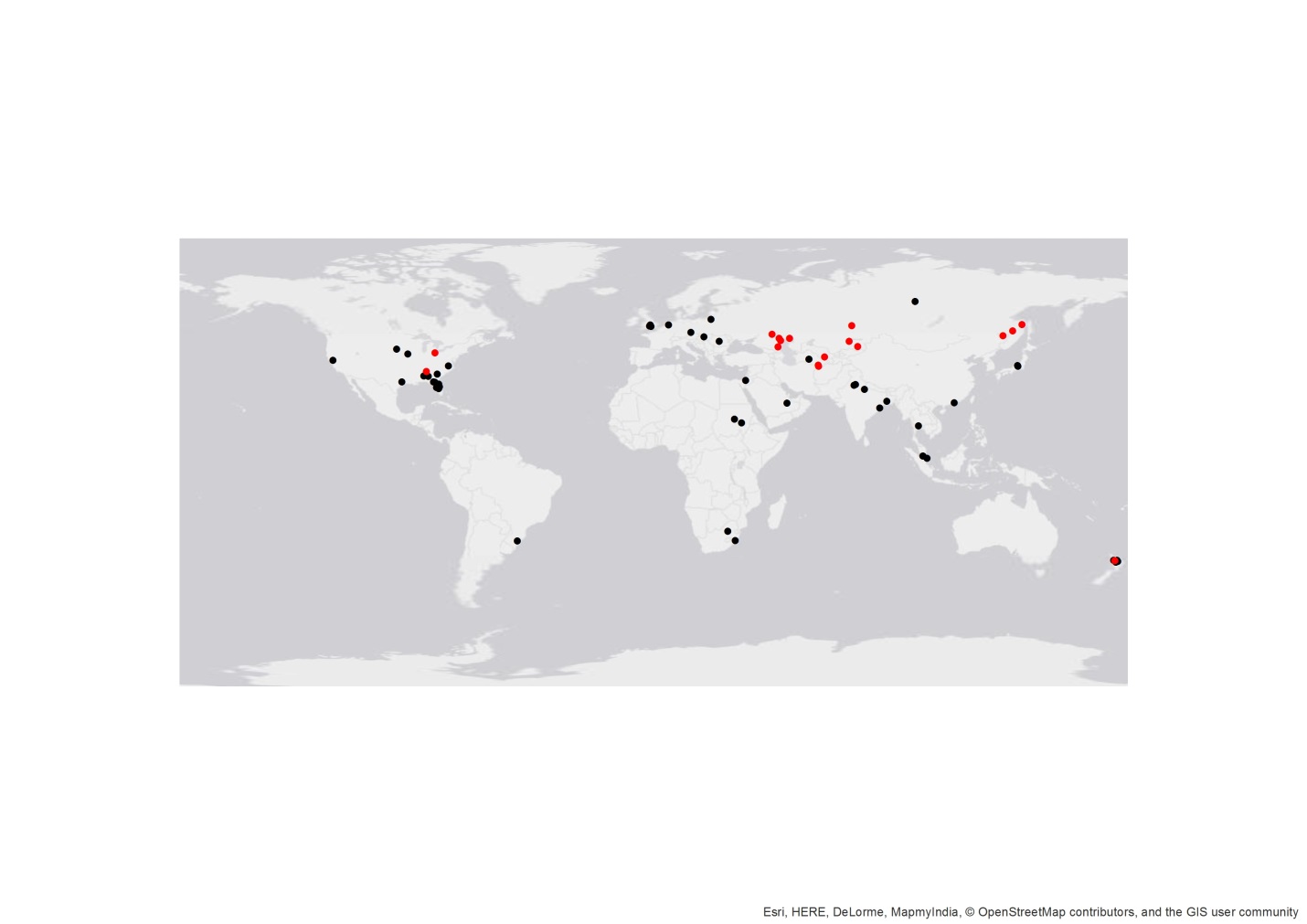


Figure B1. A subset of Grass Carp occurrence records corresponded with growth data. Such data were available for both wild (●; *n*=17) and stocked (●; *n*=51) populations. See Table B1 below for descriptions of each occurrence.

Table B1. Record of Grass Carp (*Ctenopharyngodon* *idella*) growth rate occurrences. Column headings from left to right: ID=record number, Location=Text description of Grass Carp occurrence, Lat=Latitude, Long=Longitude, Stocking rate=number of fish stocked or introduced to a system (Note: for records that indicate wild or naturalized populations, the stocking rate is either not applicable (NA) or unknown. For those records, the number of Grass Carp that were recovered from their respective ecosystem and measured in order to derive growth rates used herein is indicated in the "Stocking rate" column of the table. Experimental Period=duration of experiment or time over which growth rate is observed, Fish Age=age of Grass Carp, Wild/Stocked=Indication of Grass Carp population status, Macrophytes present=Macrophytes observed in the study system, References=Citation indicator; references included below.

| **ID** | **Location** | **Lat** | **Long** | **Stocking rate (fish/ha)** | **Experimental Period (years)** | **Fish Age (years)** | **Avg Growth (g day^-1^)** | **Wild/**  **Stocked** | **Macrophyte** | **Sources** |
| --- | --- | --- | --- | --- | --- | --- | --- | --- | --- | --- |
| 8 | Lake Wales, Florida | 27.903 | -81.584 | ? | 4.0 | 4.3 | 10.20 | s | *Hydrilla verticillata* | Shireman et al. 1980; Shireman & Maceina 1980 |
| 9 | Lake Baldwin, Florida | 28.572 | -81.322 | 24 | 2.0 | 4 | 11.48 | s | *Hydrilla verticillata* | Shireman & Maceina 1981 |
| 12 | Malaysian pond (Malacca) | 2.210 | 102.262 | 147 | 1.1 | ? | 8.32 | s | *Pennisetum purpureum* | Hickling 1960; Shireman & Smith 1985 |
| 13 | India (Cuttack) | 20.462 | 85.883 | 321 | 1.6 | 2.0 | 4.70 | s | *Hydrilla verticillata Lagarosiphon* | Alikhunhi & Sukumaran 1964 |
| 14 | India (Kalyani) | 22.990 | 88.448 | 500 | 1.0 | "Fingerling" | 13.58 | s | *Hydrilla verticillata* | Sinha & Gupta 1975 |
| 15 | India (Uttar Pradesh) | 27.638 | 80.081 | 500 | 0.5 | "Fingerling" | 9.92 | s | *Hydrilla verticillata* | Sinha & Gupta 1975 |
| 16 | India (Haryana) | 29.067 | 76.089 | 500 | 1.0 | "Fingerling" | 5.66 | s | *Hydrilla verticillata* | Sinha & Gupta 1975 |
| 21 | Hong Kong | 22.435 | 114.120 | 875 | 2.0 | 2 | 1.70 | s | Aquatic plants | Lin 1949;  Sinha & Gupta 1975 |
| 24 | Malacca, Malaysia | 2.243 | 102.258 | 1013 | 0.7 | Yearling | 5.79 | s | *Hydrilla verticillata* | Anonymous 1967 |
| 27 | Turkmenia | 38.990 | 59.020 | ? | 1.5 | 1.5 | 3.15 | s | Various | Woynarovich 1968; Sinha & Gupta 1975 |
| 28 | Rumania | 45.780 | 24.880 | ? | 1.5 | 1.5 | 1.75 | s | Various | Woynarovich 1968; Sinha & Gupta 1975 |
| 29 | Hungary | 47.490 | 19.050 | ? | 1.5 | 1.5 | 3.63 | s | Various | Woynarovich 1968; Sinha & Gupta 1975 |
| 30 | Sudan | 16.300 | 30.700 | 91 fish stocked | 3.5 | ? | 3.50 | s | *Potamogeton spp., Lemmna spp., Elodea* | George 1982 |
| 31 | Siberia | 61.012 | 99.212 | ? | ? | ? | 2.80 | s | ? | Hickling 1967;  Nikolsky 1976; George 1982 |
| 32 | Turkmenia | 38.990 | 59.020 | ? | ? | Yearling | 3.30 | s | ? | Alieve 1967;  Hickling 1967;  George 1982 |
| 33 | Israel | 31.000 | 34.870 | ? | ? | Yearling | 8.20 | s | ? | Yashouv 1957;  Hickling 1967;  George 1982 |
| 34 | Malacca, Malaysia | 2.208 | 102.261 | 12 fish stocked | 4.3 | Yearling | 9.15 | s | ? | Hickling 1967;  George 1982 |
| 36 | South Africa, Ponds at Umgeni Hatchery | -29.814 | 31.000 | 150-200 | 2.5 | Fingerling | 6.71 | s | *Vallisneria spiralis* | Pike 1977 |
| 38 | Sandusky River | 41.427 | -83.056 | NA | NA | 1.0 | 4.08 | w | ? | Chapman et al. 2013 |
| 39 | Lake Gaston, Virgina/North Carolina | 36.502 | -77.933 | ? | 5.0 | 8.5 | 3.63 | s | Hydrilla verticillata | Stich et al. 2013 |
| 40 | Gavins Point National Fish Hatchery, South Dakota | 42.850 | -97.518 | 480 | 0.4 | Yearling | 3.48 | s | *Najas guadalupensis, Chara sp., and Potamogeton pectinatus* | Harberg & Modde 1985 |
| 41 | Karma Lake, Karnal District, Haryana, India | 29.430 | 76.580 | 37 | 1.0 | ? | 20.05 | s | *Hydrilla verticillata* | Jagdish 1995 |
| 42 | Germiston Lake, South Africa | -26.231 | 28.162 | 3 | 2.0 | <1 | 7.71 | s | *P. pectinatus* | Schoonbee 1991 |
| 43 | São Jerônimo, Rio Grande, Brazil | -29.952 | -51.724 | 150 | 0.5 | ? | 0.74 | s | *Luziola peruviana* | Sponchiado et al. 2009 |
| 44 | Clay Pit III and Whittimore, Fort Gordon, Augusta, GA, USA | 33.419 | -82.140 | 80 | 1.0 | 1+ | 3.54 | s | *Eleocharis* | Terrell & Terrell 1975 |
| 45 | Waikato, New Zealand | -38.059 | 175.437 | 83 | 0.7 | ? | 10.63 | s | *Ceratophyllum demersum, Myriophyllum aquaticatum, Potamogeton spp., Plolygonum spp.* | Wells et al. 2003 |
| 46 | Suwanee Lake, Florida, USA | 28.362 | -82.230 | 299 | 1.8 | ? | 3.22 | s | *Hydrilla verticillata, Najas spp.* | Gasaway et al. 1978 |
| 47 | Broward Lake, Florida, USA | 29.512 | -81.591 | 118 | 1.8 | ? | 6.42 | s | *Hydrilla verticillata, Najas spp.* | Gasaway et al. 1978 |
| 48 | Onnaike and Maike Pond, Japan | 36.651 | 138.183 | 98 | 1.0 | <1 | 3.19 | s | *Myriophyllum spicatum, Ceratophyllum demersum, Potamogeton spp, Hydrilla verticillata, Trapa spp.* | Kurunuma & Nakamore 1957 |
| 49 | Yaits Arm, Ural River Delta, Russia | 46.884 | 51.664 | 1513 fish stocked | 8.0 | 2 | 3.63 | w | Spirogryra known to grow in this area | Tanasilchuk 1961 |
| 50 | 85 km from Astrakan, Russia | 46.892 | 47.591 | 1513 fish stocked | 9.0 | 2 | 4.51 | w | Spirogryra known to grow in this area | Tanasilchuk 1961 |
| 51 | Red Haw Lake, Iowa, US | 40.997 | -93.271 | 27 | 3.2 | ? | 5.46 | s | *Potamogeton, Najas, Ceratophyllum, and Elodea* | Mitzner 1978 |
| 52a | Waihi Beach Reservoir, New Zealand | -37.399 | 175.875 | 50-100 | 2.0 | 4 - 6 years old | 5.56 | s | *Potamogeton ochreatus, Eleocharis sphacelata, Spyrogyra spp., Zygnema spp., Mougiotia spp., Typha orientalus, Egeria densa, Nitella hookeri* | Mitchell 1980 |
| 52b | Parkinson's Lake, New Zealand | -37.315 | 174.685 | 6 to 44 | 1.0 | 5 - 6 years old | 7.29 | s | *Potamogeton ochreatus, Eleocharis sphacelata, Spyrogyra spp., Zygnema spp., Mougiotia spp., Typha orientalus, Egeria densa, Nitella hookeri* | Mitchell 1980 |
| 53 | Auburn University Ponds, AL | 32.581 | -85.497 | 74 | 0.7 | 1 to 2 | 11.00 | s | ? | Shelton et al. 1981 |
| 55 | Lake Conroe, Texas | 30.429 | -95.598 | 18 | 0.8 | 1-4 years | 7.49 | s | *Hydrilla verticillata, Ceratophyllum demersum, Myriophyllum spicatum* and 14 other species | Klussmann et al. 1988 |
| 56 | Al-Hassa, Eastern Province Agricultural Drainage System, Saudi Arabia, Drainage D-1-5 | 22.284 | 50.679 | 30000 | 2.0 | Fingerlings + 4 weeks | 3.24 | s | *Phragmites, Ceratophyllum* and *Cladophora* | Belal 2007 |
| 57a | Shinfield Lake, South Midlands, England | 51.412 | -0.938 | 221 | 1.7 | 301 g | 0.27 | s | *Myriophyllum spicatum, Potaomogeton natans, Nymphea alba, Ceratophyllum* | Fowler 1985 |
| 57b | Nether Whorton Lake, Plot A, South Midlands, England | 51.979 | -1.324 | 325 | 5.6 | 200 g | 0.40 | s | *Potamogeton crispus, Ranunculus pelatus, Sparganium erectum* | Fowler 1985 |
| 57c | Pusey Lake, South Midlands, England | 51.663 | -1.592 | 717 | 3.5 | Size range from 40 g to 500 g | 0.77 | s | *Callitriche stagnalis, Potamogeton pectintus, Vaucheria sessilis, Spirogyra spp., Tribonema spp.* | Fowler 1985 |
| 58 | Gezira, Sudan | 14.884 | 33.438 | 91 fish stocked | 3.5 | Fingerling + 2 months | 3.50 | s | *Potamogeton, Chara, Najas spp.* | George 1982 |
| 59 | Dgal Wielki, Jezioro, Poland | 54.110 | 21.793 | 15 | 1.0 |  | 1.89 | s | *Potamogeton spp., Lemmna spp., Elodea* | Krzywosz et al. 1980 |
| 60 | Vodňany, Czech Republic | 49.148 | 14.175 | 126 | 1.0 | ? | 1.37 | s | *Cladophora glomerata, Potamogeton pectinatus, Myriophyllum, Sparganium and Elatine* | Pipalova et al. 2009 |
| 61 | Suwannee Pond, Florida | 30.297 | -82.979 | 299 | 2.8 | ? | 2.06 | s | *Hydrilla verticiallata* | Gasaway et al. 1978 |
| 61 | Madison County, Florida | 30.458 | -83.507 | 52 | 2.8 | ? | 3.99 | s | *Hydrilla verticiallata* | Gasaway et al. 1978 |
| 61 | Pasco Pond, Florida | 28.222 | -82.454 | 228 | 2.8 | ? | 0.42 | s | *Hydrilla verticiallata* | Gasaway et al. 1978 |
| 61 | Broward Lake, Florida, USA | 29.513 | -81.592 | 118 | 2.8 | ? | 4.43 | s | *Hydrilla verticiallata* | Gasaway et al. 1978 |
| 62 | Davis, CA | 38.547 | -121.739 | 45 | 1.2 | ? | 2.00 | s | *Myriophyllum spicatum, Potaomogeton pectinatus, Chara sp.* | Pine & Anderson 1991 |
| 63 | National Fish Hatchery of the Beaureau of Sport Fisheries and Wildlife, Marion, Alabama | 32.633 | -87.315 | 123 | 0.5 | Fingerling | 7.36 | s | *Pithophora sp., Najas sp.* | Crowder & Snow 1969 |
| 64 | Drainage ditch in the Bay of Plenty, New Zealand | -37.701 | 176.160 | 64 | 0.6 | 2 | 0.80 | s | *Callitriche stagnalis, Nasturtium officianale* | Edwards & Moore 1975 |
| 65 | Reservoir in Ueda City, Japan | 36.400 | 138.250 | 30-100 | 2.0 | 2 | 4.29 | s | *Ceratophyllum demersum, Myriophyllum spicatum, Hydrilla verticillata, Potamogeton crispus, Trapa natans, Phragmites communis, Zizania latifolia, Scirpus fluviatilis, Glyceria acutiflora* | Tsuchiya 1979 |
| 66 | Netherlands | 51.969 | 5.665 | 285 | 0.5 | ? | 1.82 | s | *Chara, Glyceria fluitans, Alisma plantego aquatica, Callitriche sp., Polygonum amphibium, Typha sp.,* | von Zon 1973 |
| 68 | Amur River at Leninskoe (1957-1959) | 47.930 | 132.627 | NA | 1.0 | ? | 1.88 | w | ? | Smith and Shireman 1983 citing Gorbach 1961 |
| 69 | Lake Bolon and canals (1957-1958) | 49.830 | 136.370 | NA | 1.0 | ? | 0.27 | w | ? | Smith and Shireman 1983 citing Gorbach 1961 |
| 70 | Lake Udyl', Russia (1957-1958) | 52.128 | 139.855 | NA | 1.0 | ? | 1.03 | w | ? | Smith and Shireman 1983 citing Gorbach 1961 |
| 71 | Middle - Lower reaches of Terek River, Russia | 43.741 | 47.158 |  | 6.0 | 5 | 3.31 | w |  | Bogutskaya et al. citing Abdusamadov 1989 |
| 72 | Tudakul’ Reservoir, Uzbekistan | 39.856 | 64.835 |  |  | 2 | 2.96 | w |  | Bogutskaya et al. citing Nuriyev 1969 |
| 73 | Murghab River, Turkmenistan | 36.756 | 62.483 |  |  | 1 | 2.40 | w | *Phragmites australis, Myriophyllum spicatum*, and young leaves of flooded Tamarix bushes | Bogutskaya et al. citing Pavlov et al. 1994 |
| 74 | Saryyazyn Reservoir, Turkmenistan | 36.403 | 62.640 |  |  | 2.5 | 4.24 | w | *Phragmites australis, Myriophyllum spicatum*, and young leaves of flooded Tamarix bushes | Bogutskaya et al. citing Pavlov et al. 1994 |
| 75 | Balkhash Lake and the Ili River delta | 45.815 | 74.266 |  |  | 3 | 1.83 | w |  | Bogutskaya et al. citing Mitrofanov et al. 1992 |
| 76 | Chardara Reservoir, Irtysh-Karaganda reservoir | 51.817 | 75.217 |  |  | 3 | 1.14 | w |  | Bogutskaya et al. citing Mitrofanov et al. 1992 and Yakubowsky & Ereshchenko 1978 |
| 77 | Lower Volga River | 48.441 | 44.980 |  |  | 6.5 | 2.29 | w |  | Bogutskaya et al. citing Mitrofanov et al. 1992, Martino 1975, Kazancheyev 1981, Tinkovich et al. 1984 |
| 78 | Volga River Delta | 46.050 | 48.230 |  |  | 4.5 | 2.67 | w |  | Bogutskaya et al. citing Mitrofanov et al. 1992, Kushnarenko et al. 1977 |
| 83 | Kapchagay Reservoir, Kazakhstan | 43.827 | 77.516 |  |  | 2 | 0.19 | w |  | Bogutskaya et al. citing Mitrofanov et al. 1992 (referring to collections by Karpov and Glukhovtsev) |
| 84,85 | Lake Whangape, NZ | -37.469 | 175.052 |  |  | 4 | 1.36 | w |  | Baker and Smith 2006, Chisnall 1998 |

**References**

Abdusamadov A.S. 1989. Biology of perspectives of the commercial use of phytophagous fish introduced in the Daghestan Region of the Caspian Basin. [Biologiya I perspektivy rtbokhozya'stvennogo ispol'zovaniya rastitel'noyadnykh ryb, akklimatizirovannykh d dagestanskom ray'one Kaspiyskogo basseyna]. Abstracts of Candidate of Sciences Dissertation. VNIRO, Moscow. 24 p.

Alikunhi, K., & Sukumaran, K. (1964). Preliminary observations on Chinese carps in India. In *Proceedings of the Indian Academy of Sciences Section B* (Vol. 60, pp. 171–189).

Anonymous. (1967). *Report for 1967* (pp. 32–40). Batu Berendam, Malacca, Malaysia.

Bailey, W. M. (1978). A Comparison of Fish Populations before and after Extensive Grass Carp Stocking. *Transactions of the American Fisheries Society*, *107*(1), 181–206. doi:10.1577/1548-8659(1978)107<181

Bogutskaya, N. G., L. A. Jones, N. E. Mandrak, and B. Cudmore. *In prepartion.* Annotated bibliography of Grass Carp (*Ctenopharyngodon idella*) from Russian-language literature. Can. Manuscr. Rep. Fish. Aquat. Sci.:X + XX p.

Chapman, D. C., Davis, J. J., Jenkins, J. A., Kocovsky, P. M., Miner, J. G., Farver, J., & Jackson, R. P. (2013). First evidence of grass carp recruitment in the Great Lakes Basin. *Journal of Great Lakes Research*, *39*(4). doi:10.1016/j.jglr.2013.09.019

Crowder, J., & Snow, J. (1969). Use of grass carp for weed control in ponds. *Food and Agriculture Organization of the United Nations*, *2*(1), 6.

Edwards, D. J., & Hine, P. M. (1974). New Zealand Journal of Marine and Freshwater Introduction , preliminary handling , and diseases of grass carp in New Zealand. *New Zealand Journal of Marine and Freshwater Research*, *8*(3), 441–454.

Edwards, D. J., & Moore, E. (1975). Control of water weeds by grass carp in a drainage ditch in New Zealand. *New Zealand Journal of Marine and Freshwater Research*, *9*(3), 283–292.

Fowler, M. (1985). The results of introducing grass carp, Ctenopharyngodon idella Val., into small lakes. *Aquaculture Research*, *16*, 189–201.

Gasaway, R. (1978). Growth, survival and harvest of grass carp in Florida lakes. In *Symposium on culture of exotic fishes; presented at Aquaculture/Atlanta/’78* (pp. 167–183). Atlanta, GA.

George, T. (1982). The Chinese grass carp, Ctenopharyngodon idella, its biology, introduction, control of aquatic macrophytes and breeding in the Sudan. *Aquaculture*, *27*, 317–327.

Gorbach, E. I. (1961). Age composition, growth, and age of onset of sexual maturity of the White Amur Ctenopharyngodon idella (Val.) and the Black Amur Mylophryngodon piceus. *Voprosy Ikhtiologii*, *1*, 119–126.

Harberg, M., & Modde, T. (1985). Feeding Behavior, Food Consumption, Growth, and Survival of Hybrid Grass Carp in Two South Dakota Ponds. *1North American Journal of Fisheries Management*, *5*, 457–464.

Hickling, C. (1967). On the Biology of a herbivorous fish, the White Amur or Grass Carp, Ctenopharyngodon idella Val. *Proceedings of the Royal Society of Edinburgh. Section B. Biology*, (1), 62–81.

Ilan, C., & Sarig, S. (1952). Fish breeding ponds in the Far East. *Bamidgeh*, *4*(5-6), 84–111.

Jagdish, M., Rana, S., & Agarwal, V. (1995). Efficacy of grass carp (Ctenopharyngodon idella) in weed control and its growth in Karna Lake (Haryana), *27*(2), 49–55.

Kilgen, R., & Smitherman, R. (1971). Food Habits of the White Amur Stocked in Ponds Alone and in Combination with other species. *The Progressive Fish-Culturist*, *33*(3), 123–127.

Klussmann, W., Noble, R., Martyn, R., Clark, W., Betsill, R., Bettoli, P., … Campbell, J. (1983). *Control of aquatic macrophytes by grass carp in Lake Conroe, Texas, and the effects on the reservoir ecosystem*. *MP* (pp. 1–67). College Station, TX. 61: Texas Agricultural Experiment Station MP-1664.

Krzywosz, T., Krzywosz, W., & Radziej, J. (1980). The effects of grass carp, Ctenopharyngodon idella (Val.), on aquatic vegetation and ichthyofauna of Lake Dgal Wielki. *Ekologia Polska*, *28*(3), 433–450.

Kuronuma, K., & Nakamura, K. (1957). Weed control in farm pond and experiment by stocking grass carp. *Proceedings of the Indo-Pacific Fish Council*, *7*(2), 35–42.

Lin, S. Y. (1954). Chinese systems of pond stocking. *Proceedings of the Indo-Pacific Fish Council*, *5*(2-3), 113–125.

Martino K.V. 1974. Natural spawning of Grass Carp in the lower Volga system [Estestvennoye razmnozheniye belogo amura v vodoemakh Nizhney Volgi]. Gidrobiologisheskiy Zhurnal. [Hydrobiological Journal, Kiyev]. Vol. 11. No. 1. P. 91−93.

Mitchell, C. P. (1980). Control of water weeds by grass carp in two small lakes. *New Zealand Journal of Marine and Freshwater Research*, *14*(4), 381–390.

Mitrofanov V.P., Dukravets, G.M., Sidorova, A.F., et al. 1992. Annotated extracts on Grass Carp (Ctenopharyngodon idella) from: *In Fishes of Kazakhstan. [Ryby Kazakhstana] Vol. 5. Introductions, fisheries. [Akklimatizatsiya, promysel] Gylym, Alma-Ata. pp.126−159.

Mitzner, L. (1978). Evaluation of Biological Control of Nuisance Aquatic Vegetation by Grass Carp. *Transactions of the American Fisheries Society*, *107*(1), 135–145. doi:10.1577/1548-8659(1978)107<135

Morrow, J. V, & Kirk, J. (1995). Age and Growth of Grass Carp in Lake Guntersville , Alabama. *Proceedings of the Annual Conference of the Southeastern Association of Fish and Wildlife Agencies*, *49*, 187–194.

Nuriyev [Nuriev] Kh. 1969. On biology of Grass and Silver carps in the Tudakul’ Reservoir. [K biologii belogo amura i obyknovennogo tolstolobika v Tudakul’skom vodokhranilische.] Uzbekskiy Biologicheskiy Zhurnal. [Uzbek Biological Journal]. No. 6. P. 37−39.

Pavlov D.S., Aliyev D.S., Shakirova F.M., Nezdoliy V.K., Ostrovskiy M.P., Dzhemileva T.G., Malakhova T.V., Nikolayev A.A., Sukhanova A.I. 1994. Biology of fishes of the Saryyazyn Reservoir. [Biologiya ryb Saryyazynskogo vodokhranilishcha].*Gidroproekt, Moscow. 150 pp.

Pike, T. (1977). Fish and shellfish introductions; Grass carp in South Africa. *FAO Aquaculture Bulletin*, *8*(3-4), 19–20.

Pine, R., & Anderson, L. (1991a). Effect of triploid grass carp on submersed aquatic plants in northern California ponds. *California Fish and Game*, *77*(1), 27–35.

Pine, R., & Anderson, L. (1991b). Plant preferences of triploid grass carp. *Journal of Aquatic Plant Management*, *29*, 80–82.

Pípalová, I., Kvet, J., & Adámek, Z. (2009). Limnological changes in a pond ecosystem caused by grass carp (Ctenopharyngodon idella Val.) low stocking density. *Czech Journal of Animal Science*, *54*(1), 31–45.

Schoonbee, H. J. (1991). Biological control of fennel-leaved pondweed , Potamogeton pectinatus (Potamogetonaceae), in South Africa. *Agriculture, Ecosystems & Environment*, 231–237.

Shelton, W. L., Smitherman, R. O., & Jensen, G. L. (1981). Density related growth of grass carp, Ctenopharyngodon idella (Val.) in managed small impoundments in Alabama. *Journal of Fish Biology*, *18*(1), 45–51. doi:10.1111/j.1095-8649.1981.tb03758.x

Shireman, J., & Maceina, M. (1981). The utilization of grass carp, Ctenopharyngodon idella Val., for hydrilla control in Lake Baldwin, Florida. *Journal of Fish Biology*, *19*(6), 629–636.

Shireman, J., & Smith, C. (1983). Synopsis of biological data on the grass carp, Ctenopharyngodon idella (Cuvier and Valenciennes, 1844). In *FAO Fisheries Synopsis No. 135* (pp. 1–86). Rome: Food and Agriculture Organization of the United Nations.

Shireman, J. V, Colle, D. E., & Maceina, M. J. (1980). Grass carp growth rates in Lake Wales, Florida. *Aquaculture*, *19*, 379–382.

Sinha, V., & Gupta, M. (1975). On the growth of Grass carp, Ctenopharyngodon idella Val. in composite fish culture at Kalyani, West Bengal (India). *Aquaculture*, *5*, 283–290.

Sponchiado, M., Schwarzbold, A., & Rotta, M. A. (2009). Performance of grass carp (Ctenopharyngodon idella ) using Peruvian watergrass (Luziola peruviana) as main food source. *B. Inst. Pesca*, *35*(2), 295–305.

Stich, D. S., Dicenzo, V., Frimpong, E. a., Jiao, Y., & Murphy, B. R. (2013). Growth and Population Size of Grass Carp Incrementally Stocked for Hydrilla Control. *North American Journal of Fisheries Management*, *33*(1), 14–25. doi:10.1080/02755947.2012.739983

Tanasiichuk, N. P. (1961). Concerning the acclimatization of the grass carp [Ctenopharyngodon] in the lower reaches of the Volga River. *Voprosy Ikhtiologii*, *17*, 176–178.

Terrell, J., & Terrell, T. (1975). Macrophyte control and food habits of the grass carp in Georgia ponds. *International Association of Theoretical and Applied Limnology*, *19*, 2515–2520.

Tsuchiya, M. (1979). Control of Aquatic Weeds by grass carp (Ctenopharyngodon idellus val). *Japan Agricultural Research Quarterly*, *13*(3), 200–203.

Van Zon, J. (1973). Studies on the biological control of aqatic weeds in the Netherlands. In *Proc. Int. Symp. on Bioi. Contr. Weeds, 3rd, Montpellier* (pp. 31–38). Montpellier, France.

Wells, R. D. S., Bannon, H. J., & Hicks, B. J. (2003). Control of macrophytes by grass carp (Ctenopharyngodon idella) in a Waikato drain, New Zealand. *New Zealand Journal of Marine and Freshwater Research*, *37*(1), 85–93.

Woynarovich, E. (1968). New systems and new fishes for culture in Europe; Ctenopharyngodon idella, Hypophthalmichthys molitrix, Aristichthys nobilis. *FAO Fisheries Report*, *44*(5), 162–181.
